# Role of tool marks inside spherical mitigation pit fabricated by micro-milling on repairing quality of damaged KH_2_PO_4_ crystal

**DOI:** 10.1038/srep14422

**Published:** 2015-09-24

**Authors:** Ming-Jun Chen, Jian Cheng, Xiao-Dong Yuan, Wei Liao, Hai-Jun Wang, Jing-He Wang, Yong Xiao, Ming-Quan Li

**Affiliations:** 1 Center for Precision Engineering, School of Mechatronics Engineering, Harbin Institute of Technology, Harbin 150001, China; 2 Research Center of Laser Fusion, China Academy of Engineering Physics, Mianyang 621900, China

## Abstract

Repairing initial slight damage site into stable structures by engineering techniques is the leading strategy to mitigate the damage growth on large-size components used in laser-driven fusion facilities. For KH_2_PO_4_ crystals, serving as frequency converter and optoelectronic switch-Pockels cell, micro-milling has been proven the most promising method to fabricate these stable structures. However, tool marks inside repairing pit would be unavoidably introduced due to the wearing of milling cutter in actual repairing process. Here we quantitatively investigate the effect of tool marks on repairing quality of damaged crystal components by simulating its induced light intensification and testing the laser-induced damage threshold. We found that due to the formation of focusing hot spots and interference ripples, the light intensity is strongly enhanced with the presence of tool marks, especially for those on rear surfaces. Besides, the negative effect of tool marks is mark density dependent and multiple tool marks would aggravate the light intensification. Laser damage tests verified the role of tool marks as weak points, reducing the repairing quality. This work offers new criterion to comprehensively evaluate the quality of repaired optical surfaces to alleviate the bottleneck issue of low laser damage threshold for optical components in laser-driven fusion facilities.

In order to fulfill controllable fusion energy, numbers of laser beams have been focused on micro-sized target to build high-power laser systems worldwide^1^,^2^,^3^,^4^,^5^. Under such huge laser systems, a great number of large-size optical components with high-precision surfaces are required to amplify and deliver the laser beams to the vacuum target chamber. For instance, more than 30,000 pieces of optical parts are installed in the National Ignition Facility (NIF), which consists of 192 large aperture (42 cm) beams constructed by the Lawrence Livermore National Laboratory in the US^4^,^6^,^7^. Among these parts, potassium dihydrogen phosphate (KH_2_PO_4_, known as KDP) crystals are regarded as the irreplaceable components and serve as frequency converter and optoelectronic switch-Pockels cell due to their unique physical and elecro-optical properties^8^,^9^,^10^. One of the major concerns in laser fusion facilities is that under the irradiation of high-power lasers, the optical parts are susceptible to suffer from laser-induced damage, which would largely reduce their optical performances and lifetimes^2^,^11^,^12^,^13^,^14^,^15^. The laser damage on the surfaces generally threatens the laser systems more severely than the bulk damage does, since the size of surface damage would experience a rapid growth following subsequent laser irradiation, while for the bulk damage, it keeps unchanged^12^,^16^. Though the laser-induced damage on optical components has been an active field of research for over four decades, this phenomenon is still not well understood and the low laser-induced damage threshold (LIDT) remains a bottleneck in the development of high-power laser systems^17^. For KDP crystal optics, the actual LIDT is much lower than the theoretically calculated value. At this point, it is of great importance to develop new techniques for improving the laser damage resistance.

In the actual laser fusion facilities, a repair strategy has been proposed and widely applied to various terminal optics to hold the growth of unstable surface damage sites for improving the laser damage resistance. The repair strategy, which is also termed “mitigation”, is to first initiate the damage precursors at sensitive surface zones by pre-irradiation with low-fluence lasers, then identify the unstable damage sites and finally repair them with a pre-designed benign mitigation structure with much higher LIDT^3^,^12^,^18^,^19^,^20^,^21^,^22^. Two techniques of CO_2_ laser melting at 10.6 μm wavelength^3^,^18^ and ultra-short pulse laser ablation^19^ are the typical processing methods to remove the initial unstable damage sites for silica and multilayer coating optics. However, due to the delicate physical and mechanical properties of KDP crystals, micro-machining has been proven to be the most promising method to completely remove the initial damage sites on crystal surfaces^20^,^21^,^22^.

In our previous work, an efficient method by means of micro-milling tool dimpling has been proposed to repair the damage sites into spherical mitigation structure on crystal surfaces^23^. This dimpling method takes less than one minute to produce each spherical mitigation pit and the LIDT of the repaired surface is tested to be nearly three times larger than that of the initial damaged surfaces. However, in the actual repair process with cemented carbide milling tool, some tool marks would be generated inside the spherical mitigation pit owing to the flaws at the cutting edges, which may be caused by tool wear or deficiency in the tool preparation. The dimensions of these tool marks are generally comparable to the laser wavelength with several micrometers in width and sub-micrometers in depth. Surface defects with these specific sizes can largely modulate the incident laser and correspondingly lower the laser damage resistance of high-precision optical surfaces^24^,^25^,^26^,^27^. Hence, making a thorough investigation on the effect of tool marks on laser damage resistance can not only provide instructive guidance for the close supervision of tool wear in the practical repair process of KDP crystal, but also contribute to the full evaluation of the repaired crystal surfaces.

In this work, the tool marks inside spherical mitigation pit are firstly characterized by stereoscopic optical microscope. Then, the propagation process of incident laser through repaired crystal surfaces with tool marks is modeled using finite-difference time-domain (FDTD) algorithm. The light intensifications caused by mitigation pits with single and multiple tool marks are both simulated and compared with those caused by ideally repaired surfaces. Finally, the laser damage test is designed and performed for both repaired surfaces with and without tool marks to experimentally verify the role of tool marks on the laser damage resistance of repaired crystal components. Our results quantitatively demonstrate how tool marks inside mitigation pit would affect the repairing quality of damaged KH_2_PO_4_ crystal.

## Methods and Model

### Repairing damage sites on KDP surfaces by micro-milling

**Figure 1:**
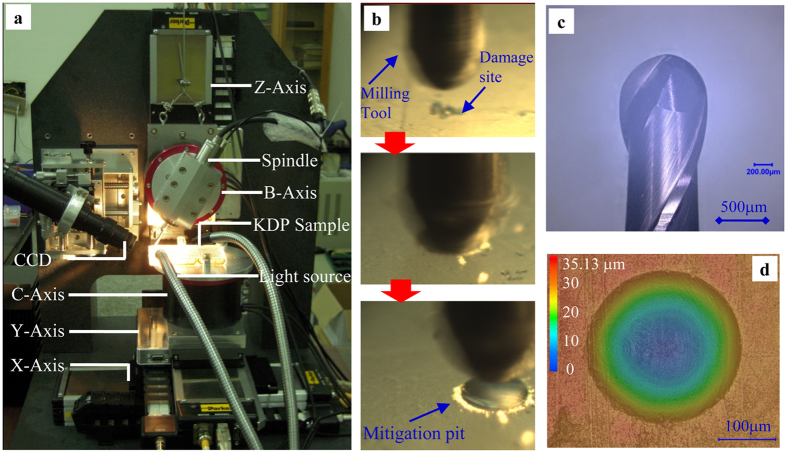
Micro-milling set-up developed for repairing the damage sites on KDP crystal surfaces. (**a**) Overall configuration of the miniature five-axis milling set-up. (**b**) The repairing process to replace the initial surface damage with a spherical mitigation pit. The initial damage site is illuminated by a cold light source and identified by a CCD camera. (**c**) The double-edged ball-end milling tool adopted in the repairing process. (**d**) The typical spherical mitigation pit without internal tool marks fabricated by micro-milling.

In the laser fusion projects, several types of specialized micro-machining set-ups have been developed to repair the unstable damage sites on high-precision crystal surfaces^12^,^20^,^21^,^22^. In order to improve the laser damage resistance of KDP crystal, we have designed and finished the construction of a miniature five-axis micro-milling set-up to fast repair the initial damaged crystal surfaces. The overall configuration and details of the milling set-up are exhibited in Fig. 1. The set-up integrates one high-speed spindle, two revolving axes (C- and B-Axis) and three linear axes (X-, Y- and Z-Axis) to achieve precision identification, positioning and repairing of the surface damage sites. The full description of the micro-milling machine can be found in ref. 23. As shown in Fig. 1b, the initial damage site is firstly detected by a CCD camera and positioned right beneath the milling tool. Then, the tiny tool, which is firmly embedded inside the spindle and rotated at high speed of 70,000 RPM, vertically feeds downward to a sufficient cutting depth, enabling the damage site to be completely removed. The remaining structure on the repaired surface is determined by the geometry of the milling tool. Figure 1c presents the double-edged cemented carbide milling tool with a radius of 500 μm, which is prepared by the NS TOOL Co., LTD (Type MSB 230) in Tokyo, Japan. The ideal fabricated spherical mitigation pit without any tool marks is shown in Fig. 1d with 240 μm width and 30 μm depth.

### Characterization of tool marks and FDTD algorithm

**Figure 2:**
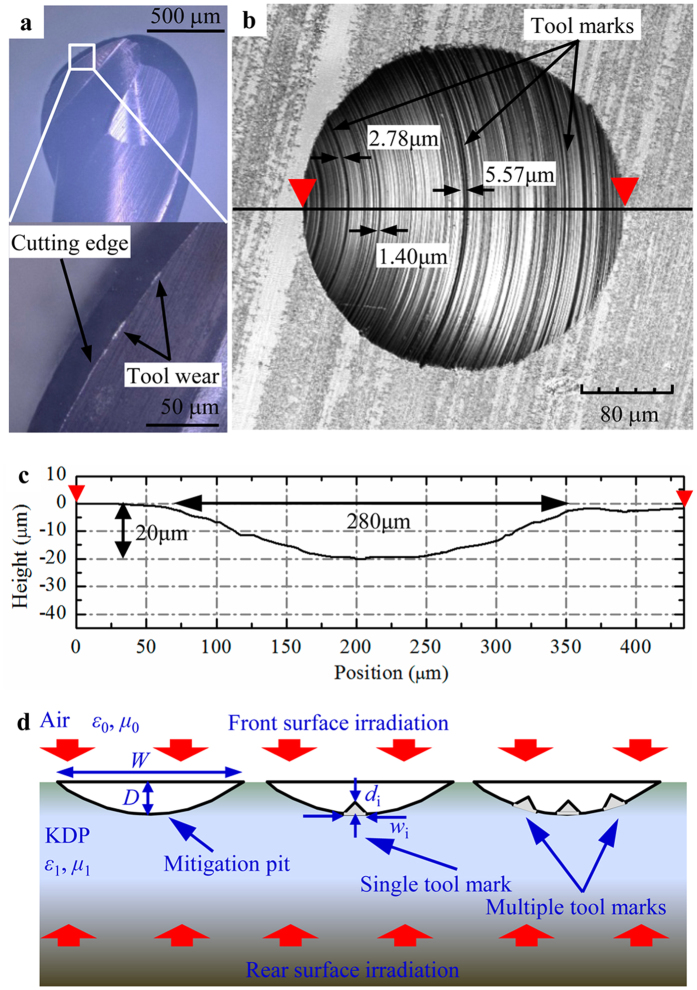
Characterization of worn milling tool edges, tool mark inside spherical mitigation pit and the FDTD simulation models. (**a**) The micro-milling tool with flaws at the cutting edge caused by wearing. (**b**) The tool marks inside repaired pit introduced by the flaws at the cutting edges. (**c**) The cross-section profile of the repaired pit with tool marks. The mitigation pit is roughly 280 μm wide and 20 μm deep, achieved using a 500 μm-radius milling tool. (**d**) Schematic of the FDTD models for ideal mitigation pit and mitigation pits with single and multiple tool marks.

In the actual repairing process with cemented carbide micro-milling tools, a series of isolated flaws can be generated at the cutting edges by means of wear and friction at the tool-chip interface^28^. As shown in Fig. 2, the cutting edges of milling tool are discontinuously chipped at some localized areas after a certain cutting period. The flaws appear to be in forms of dents or pits distributed along the edges. When the flawed tools are adopted to repair the crystal surfaces, the tool marks would be correspondingly reproduced inside the spherical mitigation pit. The reproduced internal tool marks should be like raised arc strips, which are essentially the uncut crystal material missed by the flaws at the cutting edges. The spherical mitigation pit with discontinuous tool marks are shown in Fig. 2b,c. One can see that the dimensions of the tool marks are almost several micrometers wide and sub-micrometers deep, which are both highly dependent on the wear level of the milling tool. In all the following simulations, the geometries of modeled tool marks are chosen based on the tested information above.

The modulation to incident laser light by surface features can potentially produce light intensification inside optical components, which is closely associated with the localized energy deposition and increased nonlinear absorption of intense light^23^,^24^,^25^,^29^. These negative effects introduced by tool marks would largely lower the laser damage resistance of repaired crystal surfaces. At this point, the light intensification caused by tool marks is modeled to quantitatively evaluate its bad influence on repairing quality of damaged KDP crystals. In this work, the Maxwell’s equations are numerically solved by employing FDTD algorithm to model the scenarios of light propagation through structured surfaces^30^,^31^,^32^. The FDTD models for repaired crystal surfaces with and without tool marks are presented in Fig. 2d based on the tested profiles and geometry of the practically repaired mitigation structures. For the reason that multiple tool marks can actually coexist inside the mitigation pit as shown in Fig. 2b, the effect of tool mark density is also taken into account to describe the interactions between neighboring tool marks. It has been widely known that the modulation property to incident light by rear-surface features always differs from that by front-surface structures^13^,^25^,^26^,^27^, hence, the effects of tool marks on front- and rear-surface mitigation pits are both simulated in this work. As shown in Fig. 2d, the front- and rear-surface features are realized by reversing the propagation direction in the simulations. For the sake of simplicity, the plane incident wave with TE-mode polarization and 355 nm-wavelength is adopted as the initial input source in our simulations. The electric field intensity of the initial plane wave is normalized to *E*_0_ = 1 V/m. The simulation domain is rectangular and uniformly gridded with mesh size of 25 nm, which is less than λ/12 to weaken the effect of numerical dispersion caused by differencing the Maxwell’s equations with FDTD algorithm^30^. Among all the simulations, the perfectly matched layers (PML) are employed in the vertical directions, while the periodic boundary conditions (PBCs) are applied in the horizontal directions as shown in Fig. 2d^33^,^34^. The optical parameters applied to KDP crystal and air can be found in Ref. 23.

### Laser damage resistance test

In order to experimentally testify the effect of tool marks inside spherical mitigation pits on the repairing quality, we first prepared the crystal samples with damage-free surfaces, initial damaged surfaces and repaired surfaces with and without tool marks, and then tested their respective LIDTs. The damage-free surfaces were achieved by diamond fly-cuting method and inspected with optical microscope to ensure no defect located on the surfaces. The initial damaged surfaces were artificially prepared by micro-indentation method^26^. The spherical mitigation pit with tool marks were produced by repairing the damaged crystal surfaces with worn cemented carbide milling tool as shown in Fig. 2a. To completely avoid the generation of tool mark, the repaired surfaces without tool marks were fabricated with a brand new milling tool on the basis of multiple-plunge cutting strategy, which took nearly 30 minutes to finish each repaired pit. All the features on each type of surface were further checked using optical microscope to ensure that they were in such identical shapes and dimensions that they can be applied to accurately test the statistic LIDTs.

**Figure 3:**
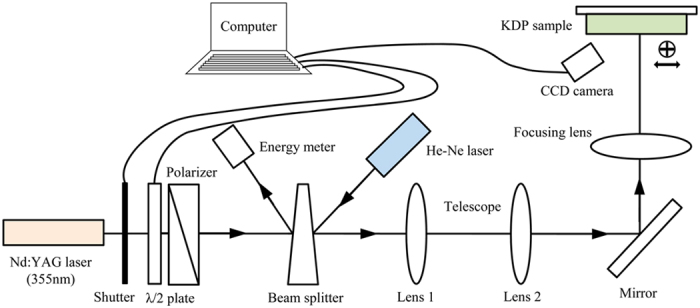
Schematic of laser damage setup for determining the LIDTs of KDP crystals.

The experimental set up for testing the LIDTs of KDP samples with various surface features is depicted in Fig. 3. The adopted Nd:YAG SAGA laser, operating at 355 nm wavelength, 10 Hz repetition rate and 6.4 ns pulse duration, is capable to output laser pulses with single longitudinal mode and approximate Gaussian spatial distribution. The 355 nm-wavelength is chosen to be consistent with that used in the simulation and actual laser fusion facilities. The switching action of laser pulses is controlled by a mechanical shutter. The combination of a wave plate and polarizer is installed following the shutter to adjust the variable laser fluence. Besides, depending on a wedged splitter, a fraction of pulse energy is detected by energy meter and a He-Ne laser is employed to calibrate the incident light. A CCD camera is placed against the KDP sample to monitor *in situ* the changes of surface morphology after each laser pulse. The LIDTs of various KDP surfaces are measured based on R-on-1 testing strategy^21^, and the detailed implementation for determining the LIDTs are described in Supplementary Fig. S1 online. It is worth noting that the laser damage experiments in this work are just designed for testing the repaired front surfaces. This is because the nonlinear property of KDP crystal makes it technically difficult to eliminate the undesirable effect of bulk damage when we attempt to test the rear surfaces.

## Results and Discussion

The light intensification associated with local energy concentration has been increasingly used by researchers to characterize the effect of surface structures on the laser damage resistance of optical components^23^,^25^,^26^,^27^,^33^,^34^. The electric and magnetic fields are simulated in this work by numerically solving the Maxwell’s equations and the light intensity enhancement factor (LIEF) is introduced to indicate the local light intensification caused by tool marks and repaired mitigation structures. The LIEF is defined as the ratio of maximum light intensity after and before the modulation of surface structures. It is worth noting that all the LIEFs caused by front-surface features are referenced to the initial light intensity in the air, while for rear-surface features, they are referenced to that inside the crystal to avoid the effect of additional reflections, which occur at the air-crystal interface^27^.

### Light intensification caused by ideal spherical mitigation pit

**Figure 4:**
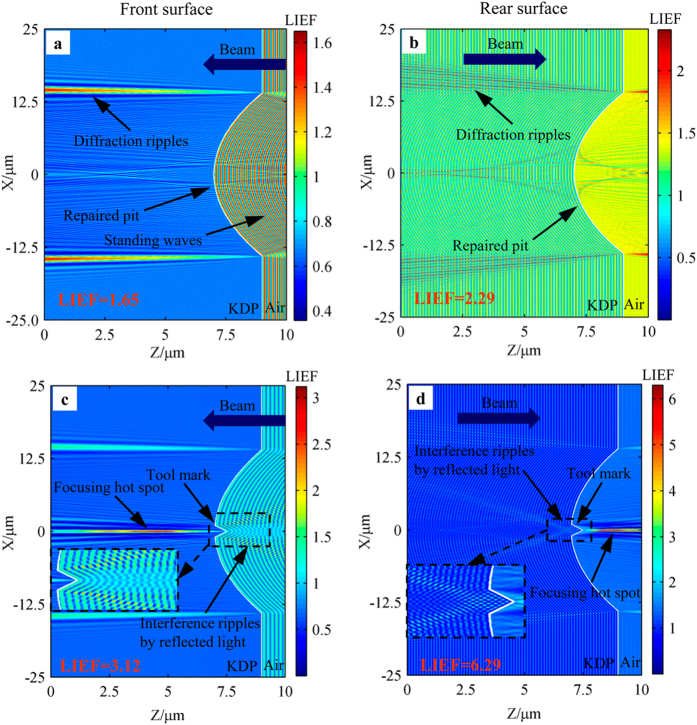
Comparison of light intensity profiles modulated by ideal mitigation pits and repaired spherical pit with single tool mark. The upper part is for the case of ideal mitigation pit on the front (**a**) and rear (**b**) surfaces, and the lower part is for the case of repaired mitigation pit with single tool mark on the front (**c**) and rear (**d**) surfaces. The tool marks in **c** and **d** are 2.0 μm wide and 0.5 μm deep. The insets are the profiles of light intensification in the vicinity of the tool marks.

In this section, the light intensification caused by ideal mitigation pit without any tool marks is simulated for making a quantitative comparison with that caused by mitigation pit with tool marks in the following sections. The model for ideal spherical mitigation pit is exhibited in Fig. 2d. Figure 4 presents the profiles of light intensification caused by ideal spherical mitigation pit on both front and rear surfaces. We should keep in mind that the reported simulation domain size is the largest dimension that we can simulate accurately using FDTD algorithm with the parameters of 355 nm-wavelength and 25 nm-mesh size. The dimension of the modeled mitigation structure is scaled down to a tenth of that of the practical mitigation structure. Nevertheless, the shape and distribution of slope along the mitigation pit contour are designed to remain the same to those of an actual mitigation pit, which can indeed provide a general trend of the light intensification caused by mitigation pit. This is because that the shape and width-depth ratio of the structure, rather than the absolute dimensions, are generally the dominant factor responsible for the light intensification caused by surface features^23^,^34^. What’s more, the primary concern of this work is to explore the negative effect of tool marks on laser damage resistance and the dimensions of tool marks throughout all the simulations are the experimental full size shown in Fig. 2. Besides, the size of the mitigation pit is roughly two orders of magnitude larger than that of tool marks. Hence, scaling down of the mitigation pit size would not affect the modulation property of tool marks, and the simulation results in the following sections would also prove it.

The profiles in Fig. 4 show that the light intensity caused by pure spherical mitigation pits without tool marks are not such largely enhanced for both the cases of rear and front surfaces. The LIEFs in the two cases are 1.65 and 2.29, respectively. However, for the initial damage site before repairing, which contains specific types of surface cracks and absorbing substance, its induced light intensification reportedly can reach up to several hundreds of times^26^,^27^. This means that repairing the initial damage site into a spherical pit is capable to greatly alleviate the light intensification and consequently improve the laser damage resistance. The slight light intensification by rear-surface mitigation pit in Fig. 4b should be blamed for the diffraction effects, originating from the intersection points of the repaired structure and the crystal surfaces. For mitigation pit on the front surface, besides the diffraction effects, the standing waves also contribute to the light intensification as shown in Fig. 4a. The diffraction profiles in Fig. 4 are consistent with the reported intensity profiles by surface contamination particles, which were calculated on the basis of Fresnel diffraction theory^35^.

### Light intensification modulated by mitigation pit with single tool mark

The single tool mark inside spherical mitigation pit is generally introduced by single flaw at the cutting edges of micro-milling tool. As depicted in Fig. 2d, the tool marks are raised uncut crystal material, which is reproduced inside the repaired mitigation pit by the chipped cutting edges. Figure 4c,d present the distributions of light intensification caused by the spherical mitigation pit with single tool mark on both front and rear surfaces. The single tool mark is set to be 2.0 μm wide and 0.5 μm deep, according to the tested geometrical information of tool marks as shown in Fig. 2. In contrary to the results for ideal spherical mitigation pit in Fig. 4a,b, the light intensification in Fig. 4c,d present quite different features. For the intensity profiles caused by front-surface tool mark in Fig. 4c, in addition to the diffraction ripples, there are two other intensified regions: focusing hot spot inside crystal and interference ripples contained in the air inside the repaired pit. The hot spot, which makes larger contribution to the light intensification, is the result of the interference of transmitted lights at the tool mark walls. This means that the raised tool mark can act as a convex lens. The LIEF in Fig. 4c is 3.12, which is almost twice as large as that caused by mitigation pit without tool mark. The other intensified region consists of interference ripples between the reflected lights from the walls of tool mark and mitigation pit as shown in the inset of Fig. 4c. It should be noted that most of the peak light intensification caused by tool marks occur inside the crystal with rare cases in which it is located in the air. For light intensification in the air, it resides very closely to the repaired crystal surface (less than 3.0 μm, as shown in Supplementary Fig. S2 online). In the actual repairing process, tool marks especially the multiple tool marks, are peculiarly prone to trap some absorbing particles (milling scraps) near the repaired surface in the air, like what the open cracks do during the finishing of silica surfaces^36^. These particles, distributed among the hot-spot regions, can strongly absorb the laser energy, and then initiate micro-explosion followed by shock waves, which would correspondingly trigger the nearby surface damage. Hence, the tool mark-induced light intensification in the air, which is close to the repaired surface, is a potential threat to the laser damage resistant, and should be taken into consideration when evaluating the negative effect of tool marks. For the case of rear-surface tool mark in Fig. 4d, the intensified regions of focusing hot spot and interference ripples coexist in the domain similar to those in Fig. 4c. The difference is that in Fig. 4d the hot spot caused by focusing of the tool mark is located in the air, and the interference ripples caused by the reflected lights at the tool mark walls reside inside the crystal. The interference ripples induced by the tool mark walls in the inset of Fig. 4d is very close to the reported results caused by rear-surface crack walls^26^,^27^. The LIEF in Fig. 4d is 6.29, which is 2.75 times as large as that caused by the ideal mitigation pit on rear surface.

**Figure 5:**
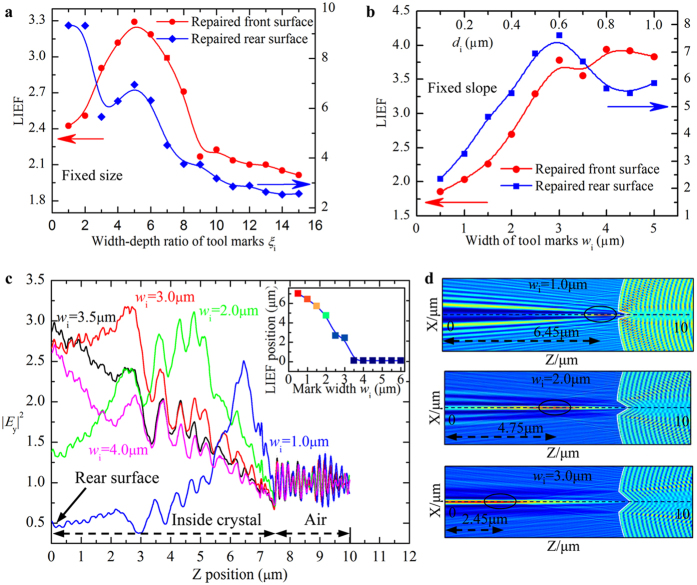
Evolution of light intensification versus structural parameters of single tool mark. The variations of LIEFs by repaired pit with single tool mark as a function of mark slope (**a**) and mark size (**b**). The mark slope (determined by width-depth ratio *ξ*_i_ = *w*_i_/*d*_i_) varies at a certain mark size (*d*_i_ = 0.5 μm), while the *ξ*_i_ keeps fixed at 2.5, when the mark size changes. (**c**) The variations of |*E*_y_|^2^ with respect to the Z position for front-surface tool marks with various widths. Z position is the horizontal distance from the rear surface (Z = 0 μm). The inset is the position of hot spot (peak intensification) with respect to mark width. (**d**) The profiles of light intensification caused by tool mark with width of 1.0 μm, 2.0 μm, 3.0 μm.

Figure 5 shows the evolution of light intensification caused by mitigation pit with single tool mark with respect to the geometrical parameters. In Fig. 5a, the slope of tool mark (determined by width-depth ratio *ξ*_i_ = *w*_i_/*d*_i_) varies at a certain mark size (*d*_i_ = 0.5 μm), while in Fig. 5b, the mark size is adjusted at a constant slope (*ξ*_i_ = 2.5). As depicted in Fig. 5a, with the increase of *ξ*_i_, the LIEF experiences a sharp increase first, then reaches a summit and finally decreases gradually. This is because, as the *ξ*_i_ increases, the angle of incidence decreases, and the transmissivity at mark walls would ascend according to the Fresnel’s reflection theory. As a result, the intensity of focusing hot spot caused by the transmitted lights presents increasing trend first. However, the position of the focusing hot spot shifts toward the rear surface as the *ξ*_i_ increases. When the *ξ*_i_ is large enough, the hot spot may reside outside the simulation domain, and the LIEF correspondingly shows a general decrease. For the rear-surface case in Fig. 5a, with increasing *ξ*_i_, the LIEF dramatically drops first, then slightly raises and gradually drops again afterwards. The competing contributions of interference ripples and focusing hot spot to the light enhancement should be responsible for this phenomenon. When the *ξ*_i_ is small, the angle of incidence is so large that the interference ripples caused by reflected lights is the dominant source, generating the largest light intensification. As the *ξ*_i_ increases, the incident angle decreases and the reflectivity drops also. Hence, the induced LIEF sharply decreases first. When the *ξ*_i_ is sufficiently large, the incident angle becomes so small that the hot spot caused by transmitted lights primarily determines the largest light intensification. As the *ξ*_i_ increases, the transmissivity increases as well, and thus, the induced LIEF shows a slight increase then. However, as the *ξ*_i_ keeps increasing, the position of the focusing hot spot would go beyond the simulation domain, leading to the final decrease of the LIEF as shown in Fig. 5a. The evolution of LIEF versus slope for given size (*w*_i_ = 2.0 μm), which shows the similar tendency to that in Fig. 5a, is presented in Supplementary Fig. S3 online. The above discussed competing effects of interference ripples and focusing hot spot can be also applied to interpret the evolution of LIEF versus the mark size shown in Fig. 5b. Nevertheless, the action of this mechanism is different in the two cases. In Fig. 5b, the increase of tool mark size would bring in the enlargement of active mark wall area, and this can affect both the amount of transmitted light and the location of hot spot. As a result, the LIEFs caused by both front- and rear-surface tool marks ascend rapidly first due to the increased amount of transmitted light, and decrease gradually for the reason of outward shift of the hot spot as shown in Fig. 5b.

From the discussions above, one can see that the shift of the hot spot position is primarily the mechanism for explaining the changing behavior of LIEF caused by single tool marks with respect to tool mark parameters. This can be further verified by the results shown in Fig. 5c,d, which exhibit the shifting behavior of focusing hot spot position caused by front-surface tool marks with various mark widths. One can see in Fig. 5c and its inset that the Z position of hot spot shifts toward the rear surface of crystal (Z = 0 μm) with the increase of mark width. When the width is large enough (*w*_i_ ≥ 3.5 μm), the hot spot would reside beyond the simulation domain, so the peak intensification keeps located at the rear surface. The shift of the focusing hot spot location is further presented in the profiles of light intensification in Fig. 5d. The shifting behavior of focusing hot spot position caused by rear-surface tool marks with various mark widths is similar to that caused by front-surface tool marks and the results are illustrated in Supplementary Fig. S2 online.

The results in Figs 4 and 5 indicate that the presence of single tool mark would lead to much higher light intensification, no matter where they dwell. The LIEFs caused by the rear-surface tool marks are generally higher than those caused by the front-surface tool marks. Among all the results, the largest LIEFs caused by rear- and front-surface tool marks are 13.4 and 3.5, respectively. This implies that the quality of repaired KDP surface can be negatively affected by single tool mark via enhancing the light intensity to 5.9 and 2.1 times (compared to those ideally repaired surfaces) for rear- and front-repaired surfaces.

### Light intensification modulated by mitigation pit with multiple tool marks

**Figure 6:**
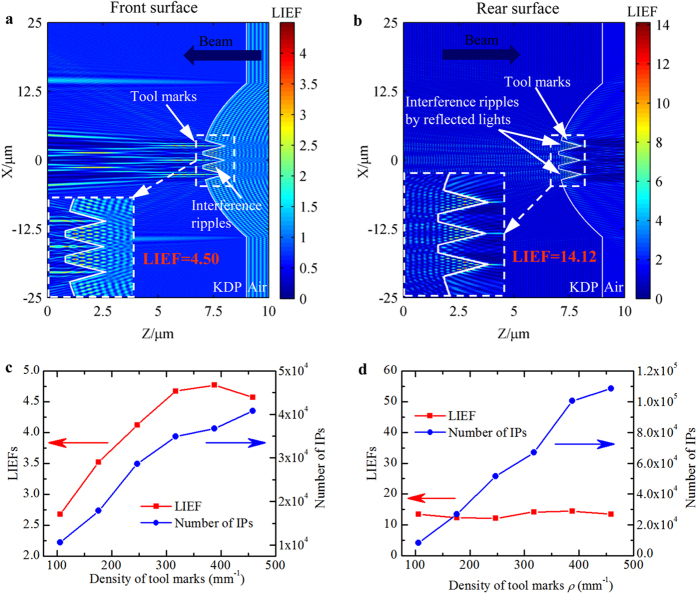
Light intensification modulated by multiple tool marks and the variations of LIEFs and number of IPs versus mark density *ρ*. Distributions of light intensity caused by repaired spherical pit with three tool marks on front (**a**) and rear (**b**) surfaces. Each of the tool mark is 2.0 μm wide and 1.0 μm deep. Insets are the profiles of light intensification in the vicinity of tool marks. The lower parts are the variations of LIEFs and numbers of IPs as a function of tool mark density *ρ* for both front (**c**) and rear (**d**) surfaces. The multiple tool marks are equally spaced, and the mark width and depth keep constant at 2.0 μm and 1.0 μm, respectively as the tool mark density changes.

In the practical repairing process, multiple flaws would be introduced simultaneously at the cutting edges and accordingly reproduce multiple tool marks inside spherical mitigation pit as shown in Fig. 2. In order to model the negative effect of actual tool marks on repairing quality of damaged KDP surfaces, it is of great importance to simulate the light intensification caused by multiple tool marks. The FDTD model for mitigation pit with multiple tool marks is exhibited in Fig. 2d. Figure 6a,b show the profiles of light intensification caused by spherical mitigation pit with three tool marks on both front and rear surfaces. The tool marks are located at the bottom of the mitigation pit with 2.0 μm-width and 1.0 μm-depth.

Similar to the case of single tool marks, the term of LIEF is applied to indicate the peak light intensification caused by tool marks. However, the laser damage susceptibility is strongly dependent on the total amount of deposited energy inside optical materials^11^,^17^. At this point, the number of intensified points (IPs) is also important to describe the laser damage resistance of repaired KDP crystal with surface structures. In this section, the number of IPs in combination with LIEF are employed to characterize the negative effects of multiple tool marks on the repairing quality. The IPs are defined as the spots, at which the LIEF is larger than that caused by ideal mitigation pit (i.e., 1.65 for front repaired surface and 2.29 for rear repaired surface). By comparing the light intensity profiles in Fig. 4 to those in Fig. 6, one sees that the incident light is much more strongly modulated by the multiple tool marks. The numbers of IPs caused by multiple tool marks are much more than those caused by single mark for both front and rear repaired surfaces. Further, compared to the identical single tool mark, the LIEF caused by front-surface multiple marks increases more largely than that caused by rear-surface multiple marks. The LIEFs caused by front- and rear-surface single tool mark are 2.7 and 13.4, as shown in Fig. 4c,d, while for multiple tool marks in Fig. 6a,b, they are 4.5 and 14.1, respectively. The new interference ripples in Fig. 6a caused by the reflected lights from neighboring tool marks should be responsible for this phenomenon.

The results in Fig. 6a,b indicate that the multiple tool marks would aggravate the negative effect on the repairing quality of damaged KDP surfaces. Based on this, we defined the tool mark density *ρ* as the number of tool mark per millimeter along the cross-sectional arc length of the spherical mitigation pit and investigate its influence on the laser damage resistance. The variations of LIEF and number of IPs with respect to the tool mark density are presented in Fig. 6c,d. It is shown that for the multiple tool marks on the front surfaces in Fig. 6c, both the induced LIEFs and number of IPs rise almost linearly as the increase of mark density. The linear rise of IPs number arises from the interference ripples caused by the reflected lights at each tool marks as shown in Fig. 6a, while for the linear rise of LIEF, it is the result of new interference ripples caused by neighboring marks. For the case of rear-surface tool marks in Fig. 6d, though the LIEF keeps roughly unchanged, the number of IPs exhibits nearly linear increase as well with the increasing tool mark density. The distributions of light intensification caused by multiple tool marks with various densities on front and rear surfaces are provided in Supplementary Fig. S4 online to further clarify the new interference ripples caused by neighboring marks and the multiplied number of IPs caused by multiple tool marks.

From the discussions above, we can conclude that, compared to single tool mark, the multiple tool marks can incur even worse negative effect on the repairing quality of KDP crystals. When the density of tool marks increases from 105.76 mm^−1^ to 246.78 mm^−1^, the number of IPs would rise up to 2.7 and 6.1 times, respectively for the front and rear surfaces. The largest LIEF caused by front-surface multiple tool marks is 1.5 times as high as that caused by single tool mark.

### Comparison of the laser damage resistances of repaired KDP surfaces with and without tool marks

**Figure 7:**
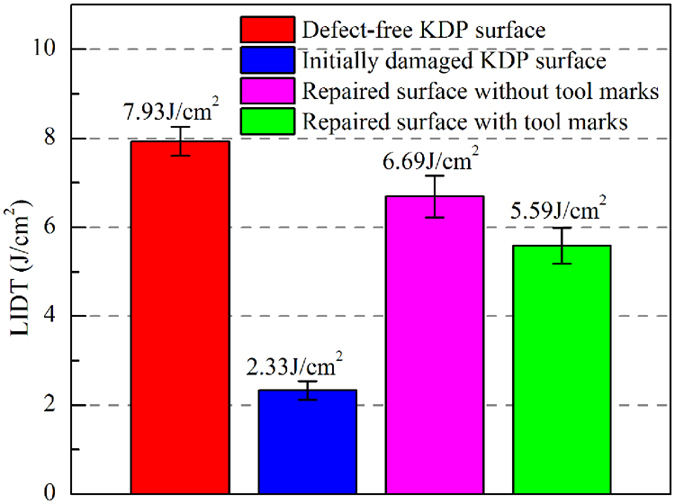
Comparison of the experimentally tested LIDTs for KDP crystals with damage-free surfaces, initially damaged surfaces, and repaired surfaces with and without tool marks. The LIDT is determined by testing 10 spots and the error bar is the standard deviation of the tested data.

The tested LIDTs of KDP crystal with different surface features are exhibited in Fig. 7. It is shown that the LIDT of diamond turned defect-free surface is 7.93 J/cm^2^. However, the growth threshold of initial damaged surface is only 2.33 J/cm^2^, which implies that once the damage occurs, the damaged surface is susceptible to suffer damage growth under the irradiation of subsequent laser pulse even with very low fluence. The LIDT of ideally repaired mitigation pit with no tool mark comes out to be 6.69 J/cm^2^. It means that replacing the initial damage site with a spherical mitigation pit by micro-milling can successfully retrieve the damage resistance of previously damaged crystal surfaces. However, with the presence of tool marks inside mitigation pit, the LIDT of repaired KDP surface decreases to 5.59 J/cm^2^, which is only 83.6% of that for ideally repaired spherical mitigation pit. The electromagnetic energy density is related to electric field **E**, 1/2 ε_0_η^2^|**E**|^2^, through the refractive index η and vacuum permittivity ε_0_^37^. As known, the light intensity is linearly proportional to |**E**|^2^. So, for a given pulse width, the laser fluence threshold LIDT is solely dependent on the light intensity. Based on this, since the tested LIDT for repaired KDP surface with tool marks decreases to 83.6% of that for ideally repaired surface, it is expected that, in the simulations, the LIEF caused by tool marks should be 1.20 times as large as that caused by ideal repaired pit. However, in the simulation results as shown in Fig. 5a and Supplementary Fig. S3 online, the LIEF caused by a front-surface tool mark with 2.0 μm-width and 0.2 μm-depth is 2.08, which is 1.26 times as large as that caused by ideal spherical mitigation pit (the LIEF caused by ideally repaired surface is 1.65). This implies that the comparison of experimentally tested LIDTs for repaired surfaces with and without tool marks agrees well with the LIEFs calculated by the FDTD simulations.

**Figure 8:**
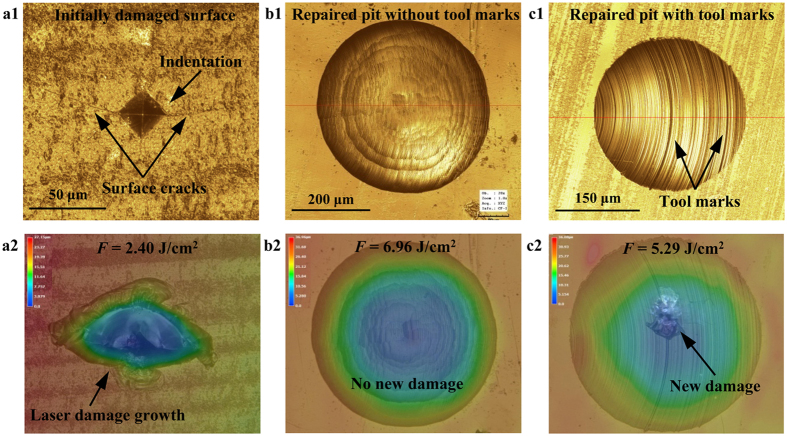
Morphologies of the various KDP surfaces before (**a**–**c**) and after (**d–f**) 355 nm-laser pulse irradiation: initially damaged surfaces (**a**,**d**), ideally repaired surfaces without tool marks (**b**,**e**) and repaired surface with tool marks (**c**,**f**). The applied fluences are 2.40 J/cm^2^, 6.96 J/cm^2^ and 5.29 J/cm^2^, respectively for initial damaged surfaces, repaired surfaces without and with tool marks.

Figure 8 presents the further experimental evidence to verify the improvement of laser damage resistance of initial damaged KDP surfaces by micro-milling repairing and the negative effect of tool marks on repairing quality. The initial damaged crystal surface with micro-indentation shows aggravated damage after the laser irradiation with 2.40 J/cm^2^ fluence as shown in Fig. 8a1,a2, while for the repaired surface without wool mark in Fig. 8b1,b2, even irradiated by laser pulse with much higher fluence (6.96 J/cm^2^), no new damage appears on the repaired KDP surfaces. However, when the repaired surface with tool marks is irradiated with 5.29 J/cm^2^ laser pulse, new laser damage comes out right at the tool marks inside the mitigation pit, which is shown in Fig. 8c1,c2. By comparing with the simulation results in Fig. 6a, it can be found that the front-surface tool marks can greatly modulate the incident light, and consequently generate a series of hot spots among the tool marks region. When incident laser comes, even with low fluence, the energy can be concentrated and multiplied in this confined hot-spot region, and hence initiate laser damage at the tool marks inside the repaired pit, which is well consistent with the actual damage scenario shown in Fig. 8c2. This implies that the tool marks are the potential weak points, lowering the laser damage resistance of repaired KDP crystals. The above experiments testify that repairing the initial damage site with a spherical pit can positively hold the damage growth for KDP optical components, while the tool marks reproduced by the flaws at the cutting edges would indeed lower the repairing quality to a certain extent.

## Conclusions

The tool marks inside spherical mitigation pit are observed when the initial damaged KDP surfaces are repaired by micro-milling. Based on the tested geometrical information of tool marks, the light intensification caused by spherical mitigation pit with and without tool marks are modeled using FDTD algorithm to quantitatively investigate the negative effect of tool marks on the repairing quality. With the presence of single tool mark, the induced LIEFs can reach up to 3.5 and 13.4, respectively for the repaired front and rear surfaces, which are 2.1 and 5.9 times as large as those caused by repaired pit without tool mark. The generation of focusing hot spots and interference ripples caused by the tool mark structures with micro/nano scales is proposed to be responsible for the aggravated light intensification. Besides, the LIEFs caused by tool marks are dependent on tool mark density and multiple tool marks can lead to much stronger modulation to the incident laser light. The numbers of IPs caused by multiple tool marks on front and rear surfaces both experience nearly linear increase with the increase of tool mark density. The LIEF caused by front-surface multiple tool marks ascend even to 1.5 times as high as that caused by single tool mark due to the interaction between neighboring tool marks. By performing the laser damage experiments for KDP samples with various surface features, it is verified that repairing the initial damage site into a spherical mitigation pit by micro-milling can largely improve the laser damage resistance and the introduction of tool marks would indeed lower its repairing quality. The LIDT for repaired crystal surface with tool marks is found be decreased to 5.59 J/cm^2^ (355 nm, 6.4 ns), which is only 83.6% of that for ideally repaired spherical mitigation pit. The experiment results are well consistent with the simulation results by combined consideration of the laser damage threshold, light intensity enhancement factor and scenarios of laser damage and hot spots. These results could provide criterion for comprehensive evaluation of the repaired optical surfaces, which are beneficial to volume fabrication and recycling of large-aperture optical components with high laser-induced damage threshold in high power laser systems.

## Additional Information

**How to cite this article**: Chen, M.-J. *et al*. Role of tool marks inside spherical mitigation pit fabricated by micro-milling on repairing quality of damaged KH_2_PO_4_ crystal. *Sci. Rep*. **5**, 14422; doi: 10.1038/srep14422 (2015).

## Supplementary Material

Supplementary Information
