# Supplementary material for: Role of tool marks inside spherical mitigation pit fabricated by micro-milling on repairing quality of damaged KH2PO4 crystal
Source: Sci Rep. 2015 Sep 24;5:14422. doi: 10.1038/srep14422 (PMC4585854; doi:10.1038/srep14422)
Supplement: Supplementary Information [file srep14422-s1.doc]

Supplementary Information for

**Role of tool marks inside spherical mitigation pit fabricated by micro-milling on repairing quality of damaged KH_2_PO_4_ crystal**

Ming-Jun Chen^1^*, Jian Cheng^1^*, Xiao-Dong Yuan^2^, Wei Liao^2^, Hai-Jun Wang^2^, Jing-He Wang^1^, Yong Xiao^1^, and Ming-Quan Li^1^

^1^Center for Precision Engineering, School of Mechatronics Engineering, Harbin Institute of Technology, Harbin 150001, China

^2^Research Center of Laser Fusion, China Academy of Engineering Physics, Mianyang 621900, China

*E-mail: chenmj@hit.edu.cn*

**1. Determination of laser-induced damage threshold (LIDT)**

In order to experimentally evaluate the laser damage resistance of repaired KDP surface with tool marks, we have tested the LIDTs for various types of KDP surfaces on the basis of R-on-1 test protocol. The detailed implementation of the R-on-1 test protocol is displayed in Fig. S1. As shown in Fig. S1, four areas with different surface features were separately prepared on the sample and a total of 10 spots on each type of area are tested respectively. The applied laser fluences are stepwise increased from initial low value *F*_0_ to *F*_0_ + *k*×Δ*F*, at which the damage occurs. The occurrence of laser damage is detected in situ by a CCD camera as shown in Fig. 3 and the LIDTs are defined as the average value of the tested fluences, at which the damage occurs. In the laser damage experiment, the pump laser should pause for 2 seconds among each pulse to eliminate the "incubation effect" caused by multiple pulses [26].


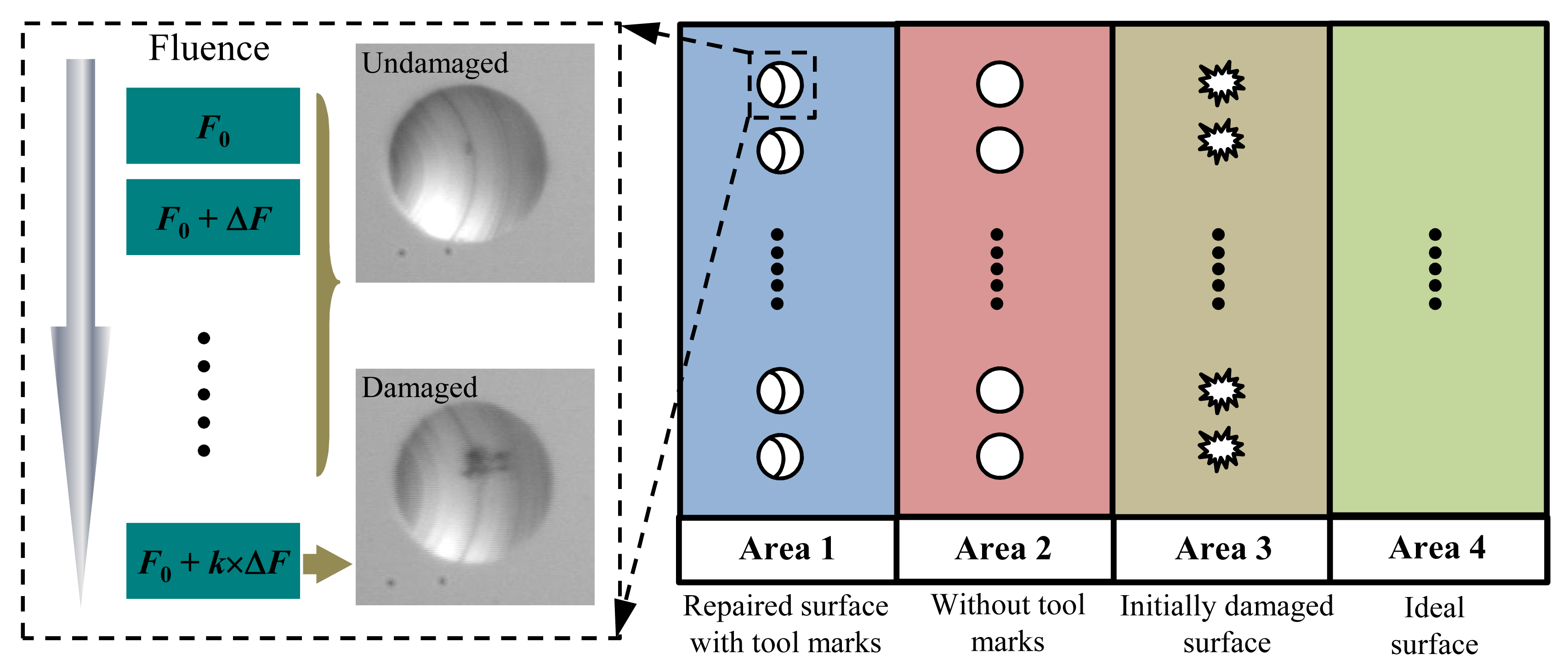


Figure S1. Details of the experimental procedures for determining the LIDTs of four types of KDP crystal surfaces. The four areas (Areas 1-4) represent repaired crystal surface with tool marks, repaired crystal surface without tool marks, initially damaged surface and ideal crystal surface, respectively.

**2. The shift of hot spot location for the rear-surface tool marks with the increase of mark width and the evolution of LIEF versus mark slope for given mark size**

The positions of focusing hot spots caused by rear-surface tool marks are also considered to reveal the underling mechanism responsible for the changing behavior of light intensity enhancement factor (LIEF) with respect to the structural parameters of tool mark as shown in Figs. 5a and 5b. The results are presented in Fig. S2 for the rear-surface tool marks with various mark widths. The positions of focusing hot spots caused by the rear-surface tool marks in Fig. S2a exhibit an evident shifting behavior toward the rear surface of crystal (Z=10μm) with the increase of mark width. When the width is large enough (*w*_i_ ≥2.5μm), the hot spot would reside beyond the simulation domain and the peak light intensification remain located inside the repaired mitigation pit at the simulation border. The shift of focusing hot spot is further described in the profiles of light intensification depicted in Figs. S2b1-S2b3. The results further verify that the shift of hot spot position is the primary mechanism for explaining the evolution behavior of LIEFs caused by single tool marks with respect to mark parameters.


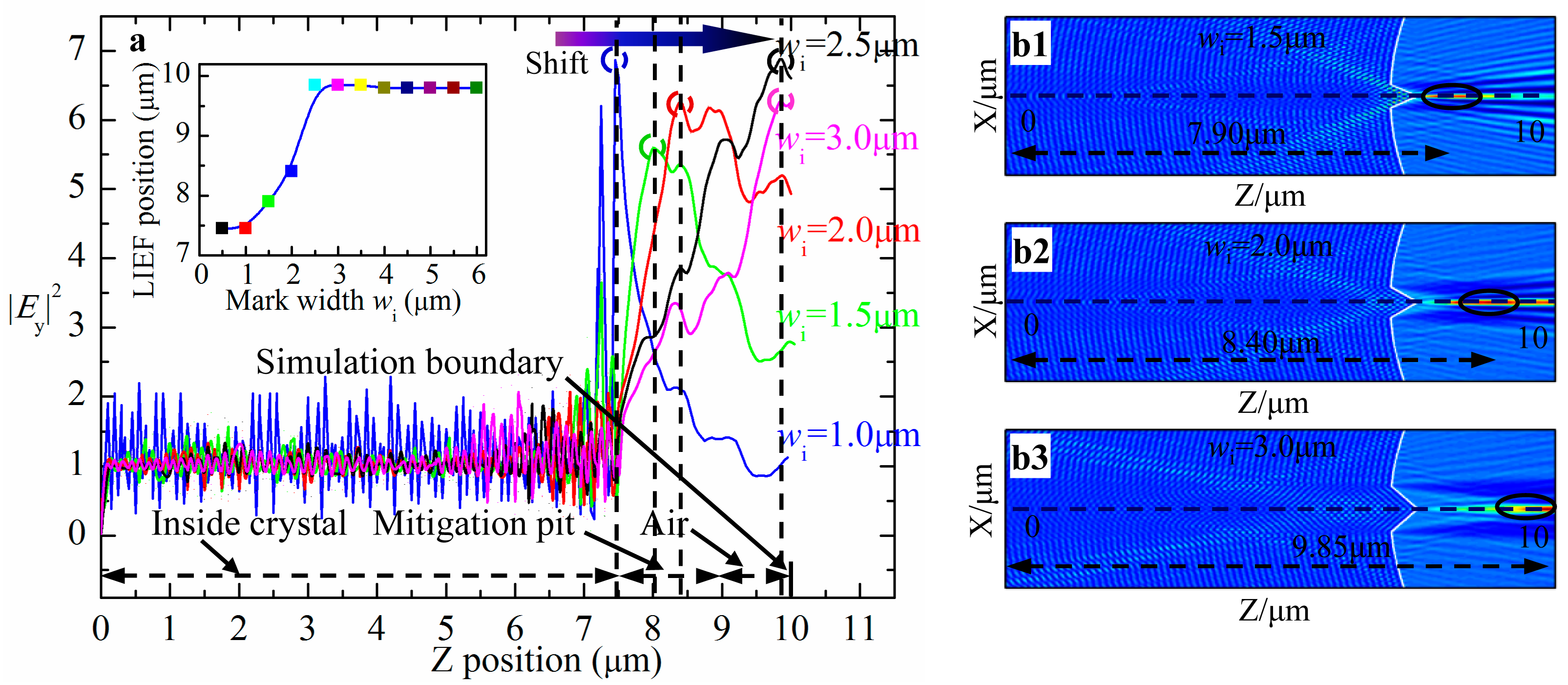


Figure S2. Simulation results of the hot spot positions for the rear-surface tool marks with various widths. **(a)** The variations of |*E*_y_|^2^ with respect to the Z position for tool marks with width of 1.0μm, 1.5 μm, 2.0 μm, 2.5 μm and 3.0 μm. The Z position is the horizontal distance from the front surface (Z = 0 μm) and the rear surface is located at Z = 9 μm. The inset is the position of hot spot (peak intensification) with respect to mark width. Figures on the right are profiles of the corresponding light intensification caused by single tool mark with width of 1.5 μm **(b1)**, 2.0 μm **(b2)**, 3.0 μm **(b3)**. The white lines in the color maps indicate the interface of air, bulk crystal and repaired surface with tool mark.

The evolution of LIEF with respect to tool mark slope (determined by width-depth ratio *ξ*_i_=*w*_i_/*d*_i_) at a certain mark size (*w*_i_=2.0μm) is illustrated in Fig. S3. The mark width keeps constant at 2.0μm as the width-depth ratio *ξ*_i_ varies. The results in Fig. S3 show the similar tendency to that in Fig. 5a, which would further verify the proposed underlying mechanism for interpreting the changing behavior of LIEF caused by tool marks with respect to mark geometrical parameters. When the mark slope changes, the competing effects of interference ripples and focusing hot spots may change, and consequently the repaired KDP crystal with tool marks would generate various magnitudes of light intensification as shown in Fig. S3.


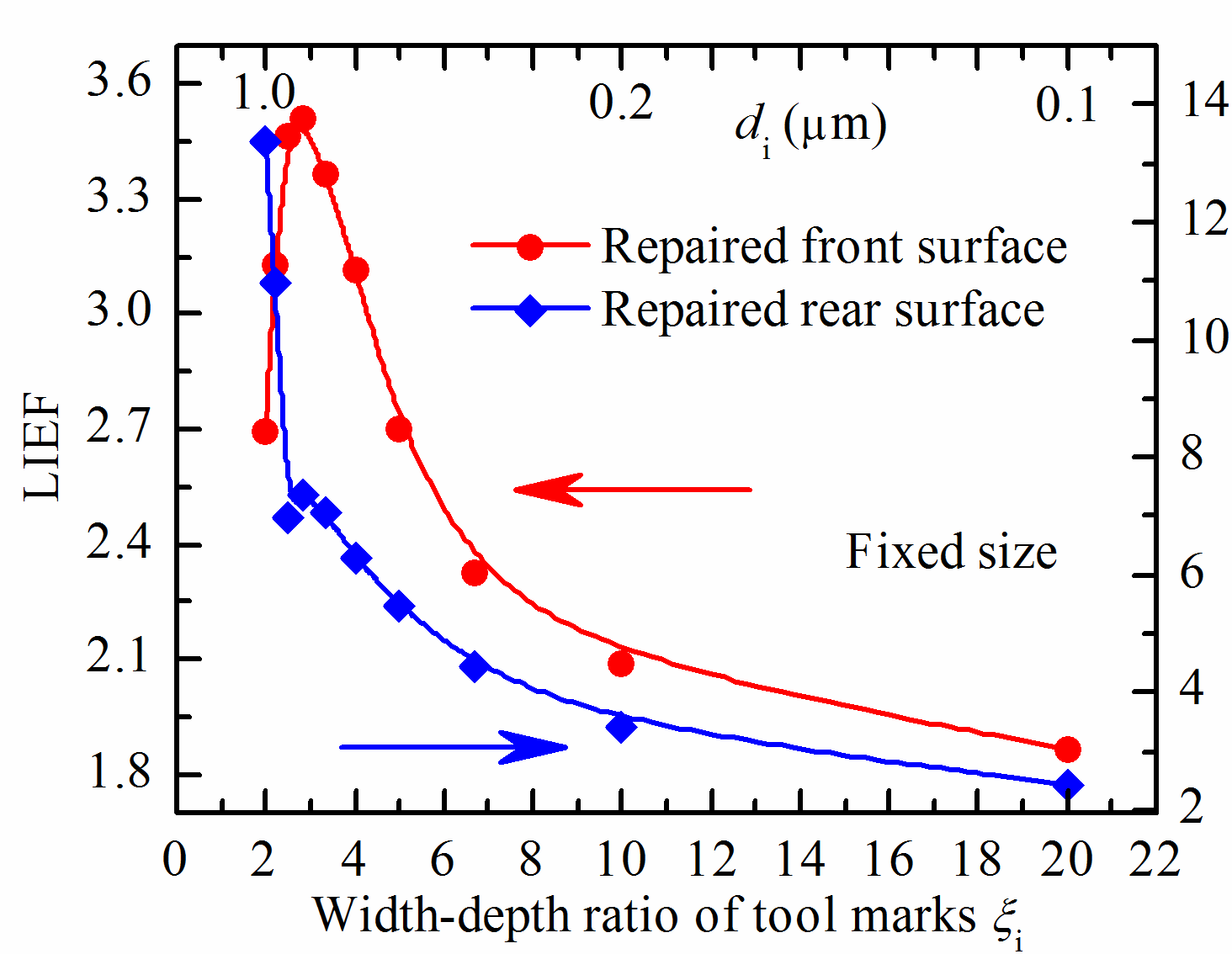


Figure S3. The variation of LIEF caused by repaired pit with single tool mark on both repaired front- and rear-surface as a function of mark slope at a certain mark size (*w*_i_=2.0μm).

**3. Light intensifications caused by multi tool marks with various mark densities**

The distributions of light intensification caused by multiple tool marks with various densities on front and rear surfaces are demonstrated in Fig. S4 to further clarify the new interference ripples caused by neighboring marks and the multiplied number of intensified points (IPs) caused by multiple tool marks. The results in Figs. S4a1-S4a3 show that the profiles of light intensification contain the focusing hot spots caused by transmitted lights at each tool mark, which is the same to those caused by single tool mark as shown in Fig. 4(c). However, new interference ripples caused by interaction of neighboring tool marks can give rise to the increase of LIEF with the increase of tool mark density. The LIEFs caused by front-surface multiple tool marks in Figs. S4a1-S4a3 are 2.68, 3.52 and 4.12, respectively. For the results of rear-surface marks in Figs. S4b1-S3b3, it is depicted that the interference ripples show up like what are observed in the case of single tool mark in Fig. 4(d). But there is no evident interaction between the neighboring tool marks. As a result, the LIEFs in Figs. S4b1-S3b3 keep roughly stable with respect to the mark density for rear-surface multiple tool marks. The profiles of light intensity in Fig. S4 also clearly show that the number of IPs caused by multiple tool marks are proportionally multiplied with the increase of mark density. The interference ripples between the incident and reflected lights at each tool mark should be responsible for the multiplication of the number of IPs.


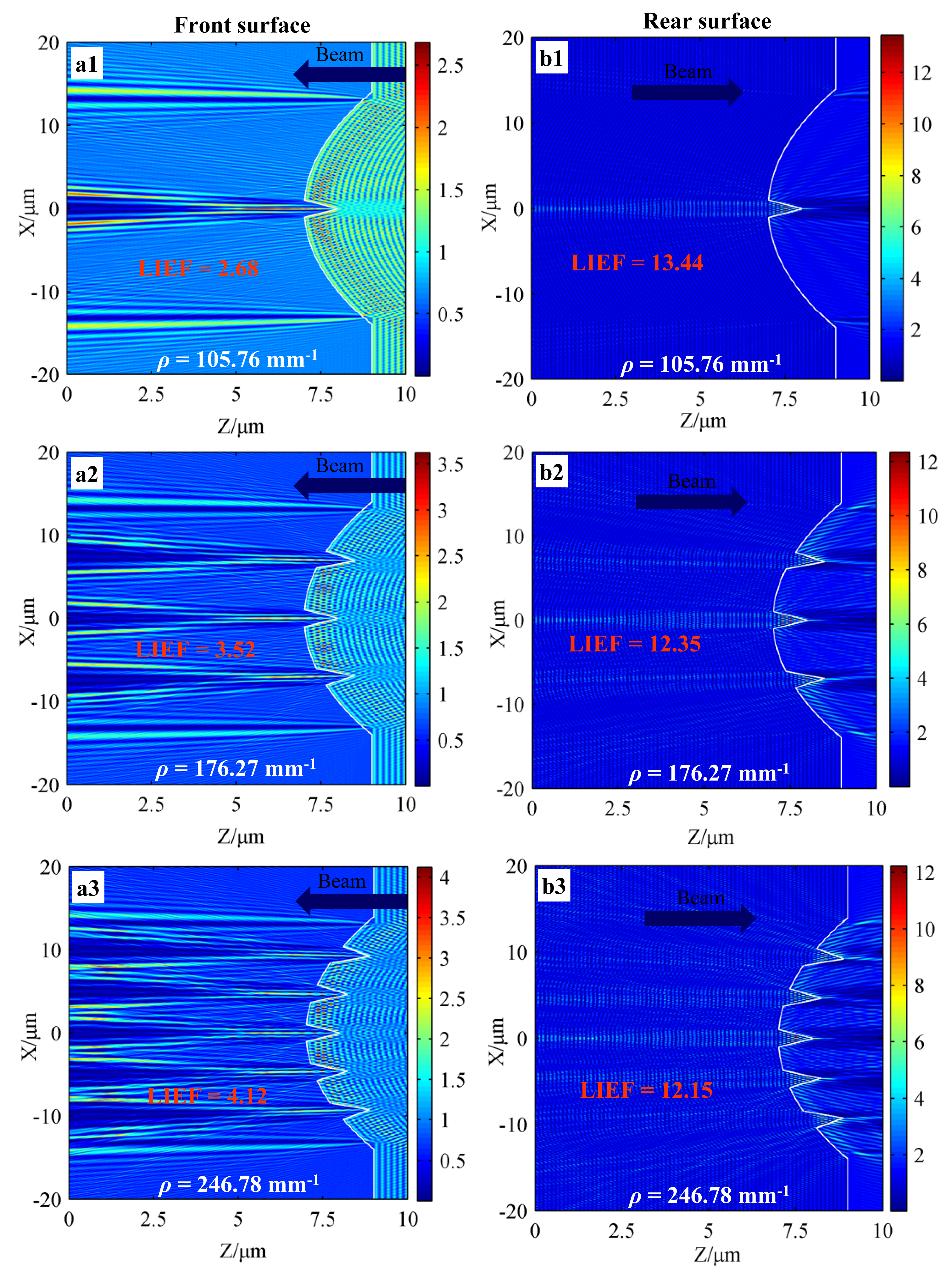


Figure S4. Distribution of light intensification modulated by both front-surface **(a1-a3)** and rear-surface **(b1-b3)** mitigation pits with multiple tool marks. The results for 2.0μm-width and 1.0μm-depth tool marks with mark density of 105.76 mm^-1^, **(a1, b1)** 176.27 mm^-1^ **(a2, b2)** and 246.78 mm^-1^ **(a3, b3)** are presented in sequence.
